# Achieving Lingualized Balanced Occlusion in a Fixed-Removable Rehabilitation for a Maxillary Complete and Mandibular Kennedy Class II Case

**DOI:** 10.1155/2019/2046421

**Published:** 2019-10-30

**Authors:** Ibrahim Tulunoglu, Samuel Cohen

**Affiliations:** Case Western Reserve University School of Dental Medicine, Department of Comprehensive Care, 2124 Cornell Rd, Cleveland, 44106 OH, USA

## Abstract

In this case report, a method to achieve an adequate compensating curve and bilateral balanced lingualized occlusion in a case requiring maxillary complete denture and mandibular Kennedy Class II removable partial and fixed prosthodontic rehabilitation is described.

## 1. Introduction

With the aging population in the advent of the baby boomers, tooth replacement is becoming more of a frequent procedure, especially with the increasing demands on patients' quality of life. Postponed or lack of replacement of missing teeth is an impending detriment to the remaining dentition. The mesial, distal, and coronal migration of unopposed teeth can be mitigated by the prosthetic restoration of edentulous spaces.

Occlusion plays a critical role in influencing the retention, stability, and the overall masticatory performance of removable prostheses. There is ample literature on lingualized, bilateral balanced, and neutrocentric occlusion [1–5], although there is no clinical evidence pertaining to the superiority of a certain tooth form or arrangement over others [6]. On the other hand, lingualized occlusion, based on a merging of esthetic and food penetration advantages of anatomic form teeth and the mechanical freedom of the nonanatomic form [3], has become more widely accepted in recent years [2].

Using a system that incorporates the tools and the artificial teeth for a lingualized tooth arrangement is useful in providing ease and consistency of the initial tooth set-up, as well as in achieving a balanced occlusion in excursive movements.

Although the methodology for fabrication of maxillary and mandibular complete dentures is well documented, it has not been well established in cases involving a maxillary complete denture (CD), opposing a mandibular removable partial denture (RPD) and fixed restorations to provide harmonious lingualized occlusion.

This paper defines a method to achieve an adequate compensating curve and bilateral balanced lingualized occlusion in a case requiring fixed and removable prosthodontic rehabilitation.

## 2. Case Report

### 2.1. Examination

A 64-year-old female presented to the DMD admitting clinic at the School of Dental Medicine with an ill-fitting maxillary acrylic-based removable partial denture. The patient's chief complaint was to get “a new set of teeth,” seeking an improvement in her esthetics and masticatory function. The patient had no major related medical conditions aside from suffering from depression and anxiety. Oral hygiene was fair and there was no periodontal disease present.

**Figure 1:**
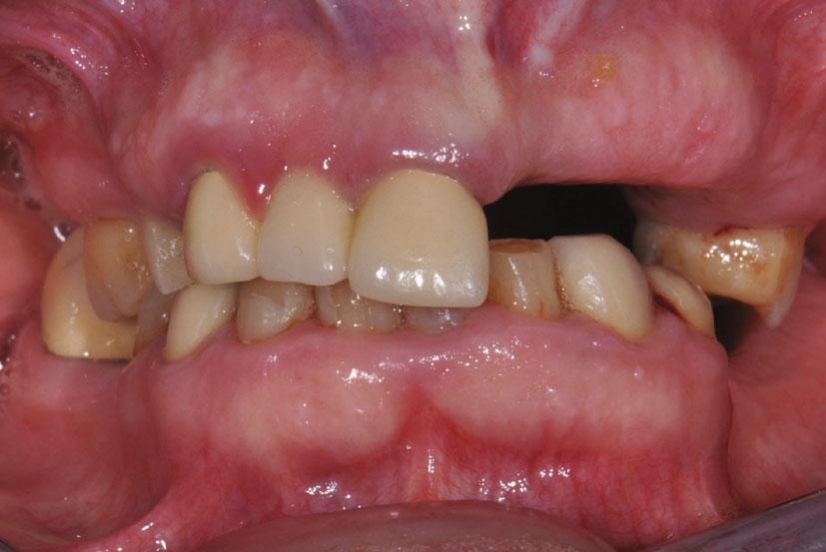
Fontal view of teeth in occlusion before treatment.

**Figure 2:**
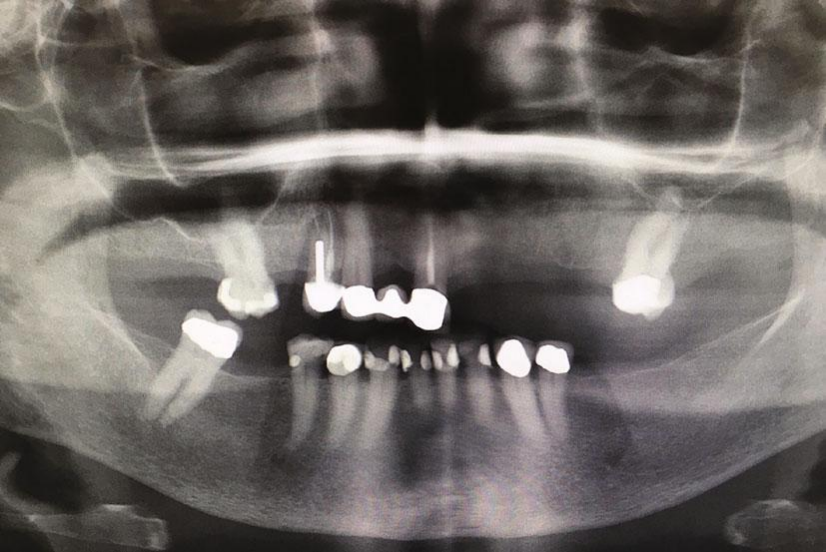
Panoramic radiograph before treatment.

**Figure 3:**
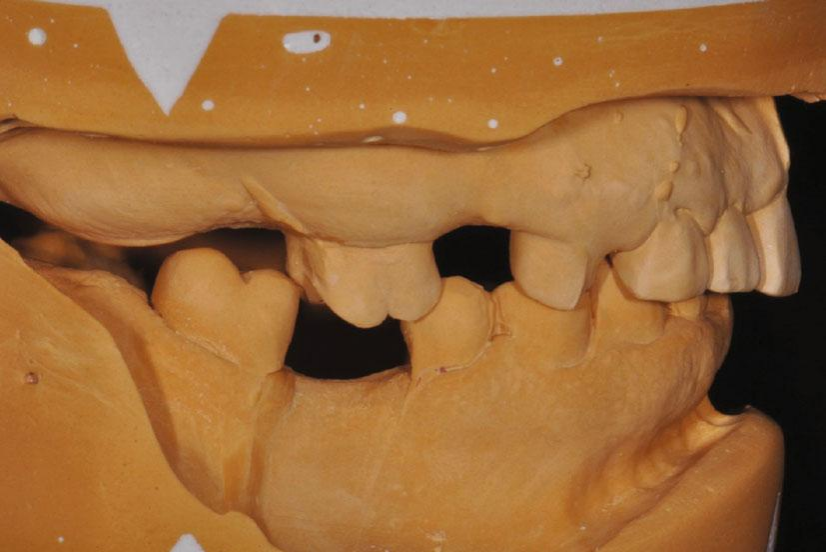
A view of mounted diagnostic casts.

Clinical and radiographic examination revealed edentulous spaces in both arches, extensive presence of nonrestorable teeth, and active caries. The patient's plane of occlusion was not harmonious, and the lack of a stable posterior occlusion led to moderate wear of her remaining anterior teeth and a collapse in her vertical dimension of occlusion which were not addressed with the previous fixed partial restorations on teeth #14, 13, and 11 (Figures 1–3). The difference between the vertical dimension of rest (VDR) and vertical dimension of occlusion (VDO) of the patient was found to be 9 mm.

After removal of the restorations, teeth #14, 13, and 11 revealed deep carious lesions, and #5 vertical root fracture. Teeth 17 and 27 showed furcation defects on the distal aspects. The patient was given various treatment plans, including full arch-supported fixed prosthodontic rehabilitation with periodontal treatment on furcation defects and crown lengthening for teeth #13, 11, 32, 31, 41, and 42 and placement of implants to support the fixed partial denture restorations replacing the tooth loss at the edentulous spans, and opted for the treatment plan involving crowning of teeth #35, 34, 43, 45, and 47 and the fabrication of a maxillary complete and mandibular removable partial denture because of the patient's avoidance from surgical placement of implants and the cost of an implant-supported rehabilitation.

### 2.2. Treatment

**Figure 4:**
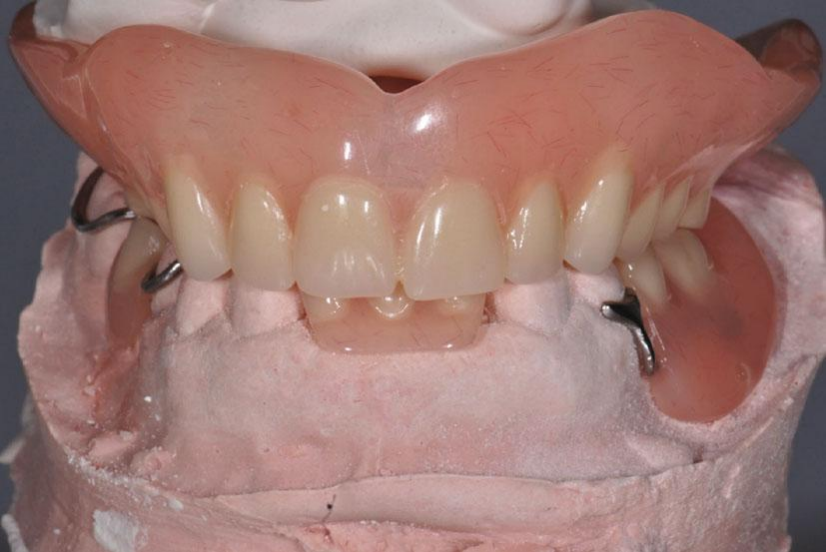
Clinical remount of interim prostheses.

After thorough oral hygiene instructions, the patient's active disease was stabilized with surgical, operative, and prosthodontic intervention. An interim maxillary complete denture and mandibular partial denture was delivered immediately after the extraction of the remaining maxillary teeth and mandibular incisors (Figure 4). In this provisional phase, the patient was rehabilitated at a 2 mm increased VDO that allowed her to be monitored in terms of any possible adverse reactions or changes in the stomatognathic system. After 6 months of osseous healing and observation period, the definitive phase of prosthetic treatment began as no adverse effect was observed in the stomatognathic system.

**Figure 5:**
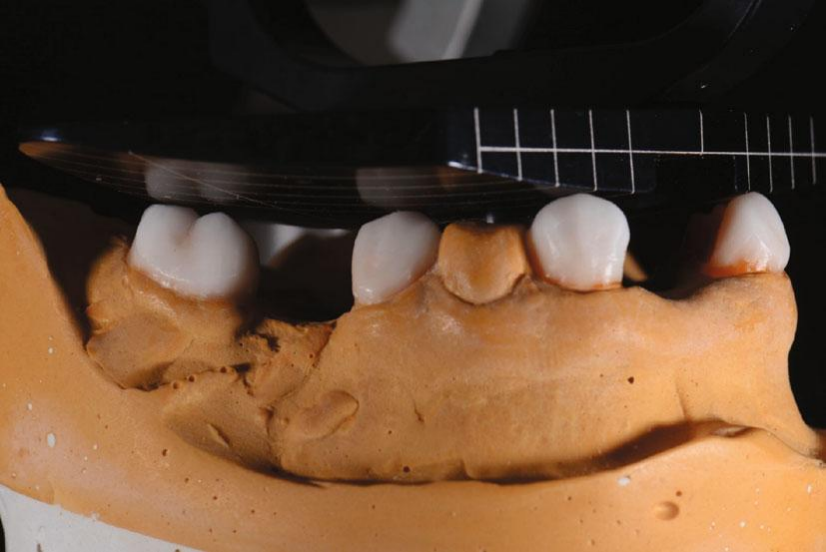
Wax-up for PFM crowns on teeth #21, 22, 27, 29, and 31.

The maxillary wax occlusal rim was adjusted intraorally to provide the correct location and position of the occlusal plane relative to the VDO lip line and smile line of the patient. Using a semiadjustable articulator (Stratos 100, Ivoclar Vivadent, Schaan, Liechtenstein), the maxillary master cast was mounted using the maxillary wax occlusal rim and the Maxillary Mounting Table (Stratos 100, Ivoclar Vivadent, Schaan, Liechtenstein) of the system. The mandibular arch was mounted in centric relation (CR). Maxillary anterior teeth (Vivadent DCL, Ivoclar Vivadent, Schaan, Liechtenstein) were set using the Mounting Table that represents the defined occlusal plane location, and the mandibular teeth were set as to obtain 1 mm of overbite and 1.5 mm of overjet. Then, the maxillary cast was removed from the upper member of the articulator, and the guide plane jig for compensating curve (2.5D Setup Template, Ivoclar Vivadent, Schaan, Liechtenstein) was inserted. The simulation of tooth preparations was made on the mandibular cast to ideal dimensions before the wax-up was made (Figure 5). An artificial tooth set-up for the maxillary posterior teeth was made to fabricate the fixed restorations accordingly.

**Figure 6:**
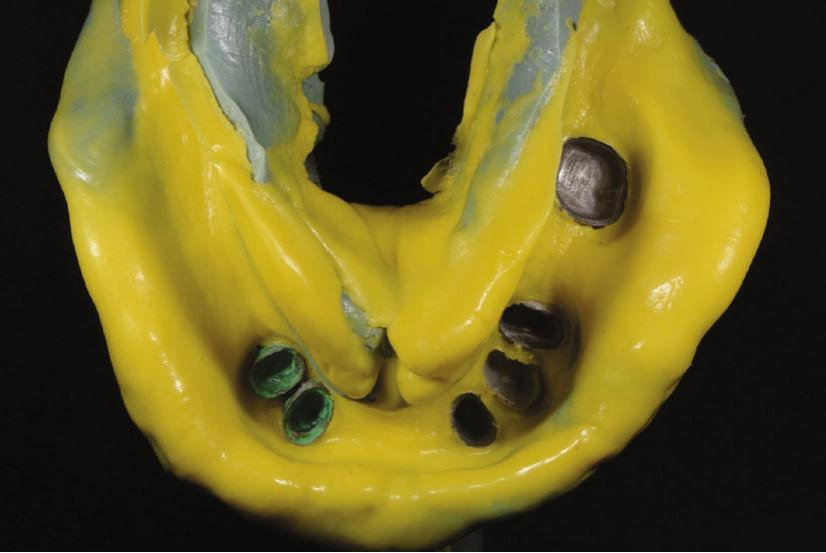
Pick-up impression of metal copings made.

**Figure 7:**
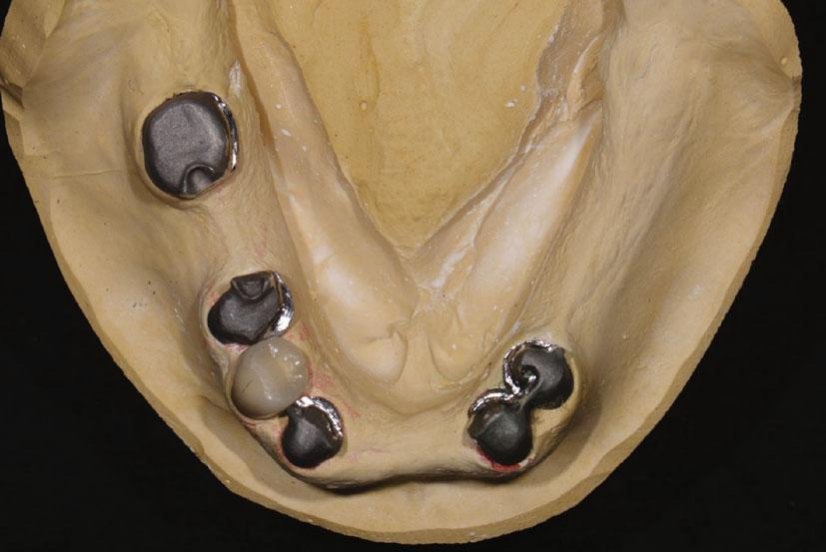
Metal copings with their exact spatial relations obtained on the master cast.

**Figure 8:**
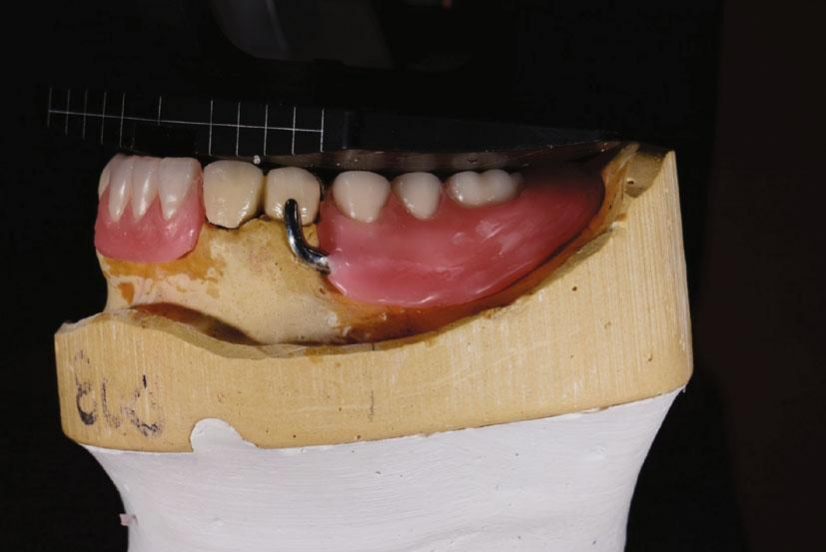
Final mandibular tooth arrangement made using the 2.5D guide plane.

**Figure 9:**
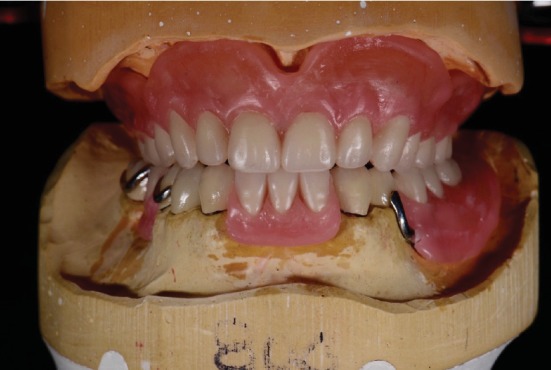
View of final tooth arrangement made.

**Figure 10:**
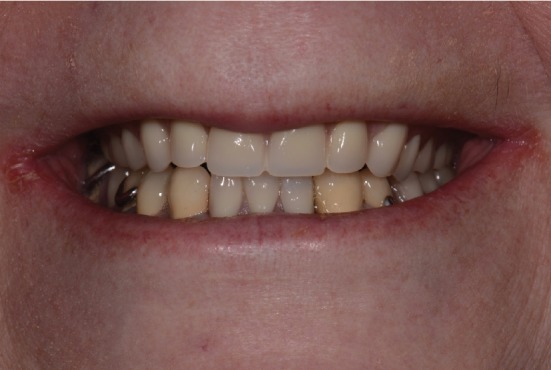
View of patient's smile at delivery.

After preparations for abutment teeth for PFM crowns #35, 34, 43, 45, and 47, the final impression was made in PVS impression material (3M Imprint 3 Heavy Body and 3M Imprint 3 Light Body). A jig for proper VDO was made on the articulator against the maxillary tooth arrangement. This jig is used as a vertical stopper for the intermaxillary relationship record to replicate the VDO. Surveyed crown restorations with guide planes parallel to the defined path of insertion and ledges were made, to improve retention, stability, and support of the RPD and tried intraorally. A coping pick-up impression was made with the PVS impression material (Figure 6) and poured with stone (Microstone, Whip Mix Corp.) (Figure 7). The guide planes and gingival ledges were refined and milled once more, including the dovetail-shaped rests between the splinted copings on 21 and 22. Then, porcelain was built on the copings following the 2.5D template and the maxillary tooth arrangement was also made according to the same template (Figure 8). After the crowns were made, the RPD metal framework was fabricated for the Kennedy Class II mod II mandibular arch. The metal framework and the crowns were tried in together, and the final tooth arrangement was made with the same template (Figure 9). Final try-in of the maxillary CD, mandibular RPD, and fixed prostheses allowed for minor adjustments in the tooth set-up. The final tooth arrangement in wax shows the harmonious occlusion between the fixed and removable elements of the prostheses. At delivery, minimal occlusal adjustments were done intraorally to achieve lingualized occlusion with balanced centric and eccentric contacts (Figure 10).

## 3. Discussion

With such a dilapidated dentition and loss of vertical dimension, it was of utmost importance to restore the patient's occlusal plane so that proper function could be obtained. Not only is having proper centric contacts on all teeth essential for function, but having those contacts orientated in the correct occlusal plane and compensating curve was imperative. Extractions, surveyed metal-ceramic restorations with parallel guide planes, a mandibular removable partial denture (RPD), and maxillary complete denture (CD) were treatment avenues used to rehabilitate this patient. An approach utilizing the Stratos 100 Articulator and 2.5D guide plane was sought to harmonize the occlusion between the fixed and removable elements of the mandibular prostheses.

A concern that will inherently come with any system that does not include a face bow transfer may be whether this would cause a big discrepancy between a patient stomatognathic system and articulator. Kumar et al. compared two methods, one with and one without face bow transfer and concluded that balanced occlusion can be achieved successfully with a system that does not require face bow transfer [7]. In this clinical case, we did not observe a difference between the interarch relationship in the patient's mouth and the articulator. The system allowed to establish the desired occlusal plane location and position as well as the contact patterns that are designed as part of the standards established with the Stratos system. This included establishing the occlusal configurations as well as the positions and locations of both fixed restorations and artificial teeth on removable dentures with minimal adjustment at delivery. Instead of maxillary and mandibular posterior teeth with steep cuspal angles, using artificial teeth fabricated purposely for lingualized occlusion also contributed to the fact that fewer adjustments overall necessitated.

The *Glossary of Prosthodontic Terms* defines the compensating curve as “the anteroposterior curving (in the median plane) and the mediolateral curving (in the frontal plane) within the alignment of the occluding surfaces and incisal edges of artificial teeth that is used to develop balanced occlusion” [8]. A balanced occlusion is desirable for all removable complete denture rehabilitations. In other words, this is one of the five major determinants of balanced occlusion expressed in Hanau's Quint and Thielemann's formula [2, 3]. The compensating curve is provided by the 2.5D template, and the cusp heights are also provided by the artificial teeth set produced specifically for lingualized occlusion.

## 4. Conclusion

Using the principles of lingualized occlusion in complete dentures, it is possible to achieve lingualized balanced occlusion in a maxillary edentulous and mandibular Kennedy Class II fixed-removable rehabilitation case.
